# Proliferating and differentiating effects of three different growth factors on pluripotent mesenchymal cells and osteoblast like cells

**DOI:** 10.1186/1749-799X-2-27

**Published:** 2007-12-20

**Authors:** Britt Wildemann, Nicole Burkhardt, Marc Luebberstedt, Thomas Vordemvenne, Gerhard Schmidmaier

**Affiliations:** 1 Center for Musculoskeletal Surgery, Charité-Universitätsmedizin Berlin, Germany; 2 Berlin-Brandenburg Center for Regenerative Therapies, Berlin, Germany; 3 Dept. Trauma, Hand and Reconstructive Surgery, University Hospital, Muenster, Germany

## Abstract

Growth factors are in clinical use to stimulate bone growth and regeneration. BMP-2 is used in long bone and spinal surgery, PDGFbb for the treatment of periodontal defects and children with growth hormone receptor deficiency are treated with IGF-I.

Aim of the present study was the comparative analysis of the effect of these growth factors released from a local drug delivery system on cells of the osteogenic lineage at differing differentiation stages.

The experiments with the mesenchymal cell line C2C12 revealed a proliferating effect of all three growth factors and a differentiating effect of BMP-2 with a dramatic increase in alkaline phosphatase activity. None of the growth factors stimulated cell migration.

Human osteoblast like cells showed similar results with an increase in proliferation after stimulation with IGF-I or PDGFbb. The enzymatic activity of alkaline phosphatase was enhanced only in the cells stimulated with BMP-2. This group showed also more mineralized matrix compared to the other groups.

In conclusion, the growth factors IGF-I and PDGFbb delivered with a local drug delivery system stimulated cell proliferation, whereas BMP-2 showed a dramatic effect on differentiation on osteoblast precursor cells and osteoblast like cells.

## Background

Today BMPs are used in spine and orthopaedic surgery, the platelet derived growth factor (PDGFbb) for periodontal treatment [1] and insulin growth factor-I (IGF-I) to treat children with growth hormone insensitivity syndrome or IGF-I deficiency [2,3]. These three growth factors belong to different families and initiate their signaling from the cell surface by different receptors and intracellular pathways. The IGF and PDGF signals are transduced via tyrosine kinases [4,5] and the BMP signal via serine/threonine kinase [6,7]. Several *in vitro *and pre clinical *in vivo *studies have been performed to demonstrate the effect of the growth factors on different cell types and bone [8-13].

For the clinical use of growth factors the delivery system is important [14]. Once a growth factor reaches the site of action, it must remain at the site in an appropriate concentration and long enough for the pharmacological effect. The half life of growth factors *in vivo *is very short and they are metabolized within a few hours [15]. For the use in bone regeneration, however, an action over a longer time period is necessary. For a therapeutical success these requirements must be met and therefore an adequate carrier must be used for drug delivery. A drug delivery system based on poly(D,L-lactide) (PDLLA) was developed for local release [16]. In previous studies the release profile of the growth factors incorporated in the PDLLA coating by eluting in PBS or cell culture medium was investigated. The incorporated growth factors were released with an initial peak with in the first 2 to 3 days. The peak release is followed by a slow sustained release [16,17]. Storage of the coated implants over 14 month had no effect on the activity of the incorporated growth factors on osteoblast like cells [17]. The PDLLA serves as a coating for orthopedic implants with incorporated pharmacological agents. Using this application system, aim of the study was the comparison of BMP-2, IGF-I and PDGFbb in their effect on different cell types. Primary human osteoblast like cells were used to investigate the effect of the growth factors on bone forming cells. The used cell line C2C12 differentiates rapidly into myoblasts after reaching confluence [18]. This cell line has also the potential to differentiate to adipocytes [19] or osteoblast like cells [18] and therefore serve as a model for pluripotent mesenchymal cells. The potential of these cells to differentiate into the osteoblastic linage is used to test the osteoinductivity of bone grafting materials [20]. Using the C2C12 cells the osteoinductivity and migratory effect of the growth factors was analyzed.

## Methods

### Cell culture

Osteoblast like cells were isolated from tibia plateau after reconstructive surgery with the permission of the local authorities. An informed consent was obtained from all donors. For isolation of the cells, the trabecular bone was minced into little pieces followed by overnight digestion with collagenase Type II according to established protocols [21] 1 × 10^5 ^osteoblasts were cultivated in 12 well plates in DMEM/F-12-media with 10% heat inactivated FCS at 37°C and 5% CO_2_. After cultivation of the cells for 3 days under identical conditions, the implants were added to the culture in a non-contact manner using a tissue culture inserts (0.4 μm pore size, Nunc, Germany). The cells were cultured for further 15 days. One third of the medium was changed every day to ensure only gentle changes in the medium composition and growth factor concentration.

Three parallel test series were performed with pooled cells from different donors. Each test series was done in triplicate.

The mice myoblast cell line C2C12 (ACC 565) was obtained from DSMZ, Braunschweig, Germany. 5 × 10^4 ^cells were cultivated in 24 well plates in DMEM with 10% heat inactivated FCS at 37°C and 5% CO_2_. After a 5 h adherence period medium was changed to DMEM with only 1% heat inactivated FCS to reduce the proliferation activity. The implants were placed into the culture wells and the cells were cultured for three days. The test were conducted in triplicate and repeated two times.

### Growth factors

The growth factors were applied to the cell culture from a local drug delivery system. The drug delivery system is based on a Poly(D,L-lactide)-coating (Boehringer, Ingelheim, Germany) on Titanium Kirschner-wires (1.0 mm diameter, Synthes USA) and described in more detail elsewhere [16].

Three different recombinant human growth factors were used for the experiments:

IGF-I (R&D-Systems, Wiesbaden, Germany), BMP-2 (Osteogenetics, Würzburg, Germany) and PDGFbb (Biomimetics, Franklin, USA).

According to previous experiments [11,12] the growth factors were incorporated in 5% (w/w) in the PDLLA coating. The amount of growth factor added to the cell cultures was 15 μg/ml (osteoblast) and 10 μg/ml (C2C12).

The difference in the applied growth factor amount (15 μg/ml or 10 μg/ml) is due to the different cell culture approaches (24 well plates or 12 well plates) used and the fact that the factors were applied from coated titanium k-wires. For control served k-wires coated with the carrier PDLLA.

### Analysis

Cell vitality and proliferation was achieved via a non invasive/toxic cell activity assay (alamarBlue, Assay, Biozol, Eching, Germany). For the assay, 10% alamarBlue was added to the cells and incubated for 3 h at 37°C. The absorbance was measured in triplicate spectrophotometrical with a micro plate reader at two wavelengths: 570 and 600 nm in accordance to the instruction of the manufacturer.

The catalytic activity of the alkaline phosphatase (AP) was determined using para-nitrophenyl phosphate (p-NPP, Sigma, Germany) as a substrate of the enzyme. After rinsing the cells the freshly prepared AP-buffer was added and incubated for 30 min at 37°C. The absorbance was read out in triplicate on a micro plate reader by 405 nm wavelength.

The Osteocalcin concentration was quantified with an ELISA (Metra Osteocalcin EIA kit, Quidel, San Diego, CA). De Novo synthesis of collagen type-I was quantified to detect carboxyterminal propeptide of type I collagen (CICP, Quidel, San Diego, CA), a decomposition product of collagen type-I. Supernatant from the cell cultures was used and both ELISA were performed in accordance to the instructions of the manufacturer.

Matrix mineralization was evaluated by the use of Von Kossa stain. Cells were rinsed and fixed with cool methanol for 10 min. After rinsing with water cells were incubated for 30 min at room temperature (RT) with 3% silver nitrate and for 2 min in formaldehyde. Surplus silver nitrate was removed by incubation in 5% sodium thiosulfate for 5 min at RT.

Migration assay was performed by using a well established assay [22]. Briefly, the growth factor coated wires were placed to the lower chamber of polycarbonate-membrane-inserts (8 μm pore size, Nunc, Germany) and covered with DMEM with 0.1% BSA. After 24 h 5 × 10^4 ^pluripotent mesenchymal cells (C2C12) were added to the upper chamber and cultured under standard conditions. For positive control, 10% FCS was added to the lower chamber. After 5 h of incubation, the membrane was removed and the cells on the surface carefully abscised. Cells migrated into the membrane were fixed with 4% w/v paraformaldehyd (PFA, Sigma, Germany), stained with 4',6-Diamidino-2-phenylindole (DAPI, Sigma, Germany) and counted under the microscope.

### Statistical analysis

In order to compare the data of the independent test serials, the results of the experimental groups were normalized to the results of the PDLLA-group (control).

Statistical differences were assessed using an ANOVA and Dunnett Post Hoc test was employed for multiple comparison tests at a level of 95% (Software SPSS12.0).

## Results

### Osteoblast like cells

**Figure 1:**
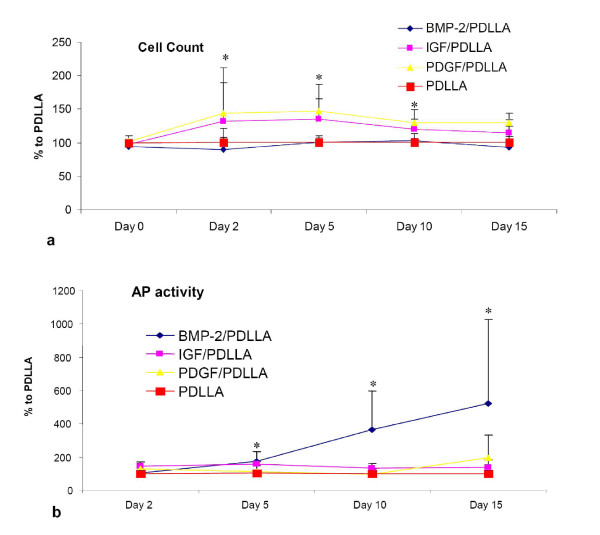
a) Cell count of the osteoblast like cell culture treated with different growth factors. The data presented are normalized to the control group (PDLLA) which is set 100%. A significant increase in the cell number was seen after treatment with PDGFbb or IGF-I (days 2–10) in comparison to the PDLLA treated cells (ANOVA, Dunnett). b) Alkaline phosphatase activity (AP) of the osteoblast like cell culture treated with different growth factors. The data presented are normalized to the control group (PDLLA) which is set 100%. A significant increase in AP activity was seen after treatment with BMP-2 (days 5–15) in comparison to the PDLLA treated cells (ANOVA, Dunnett).

**Figure 2:**
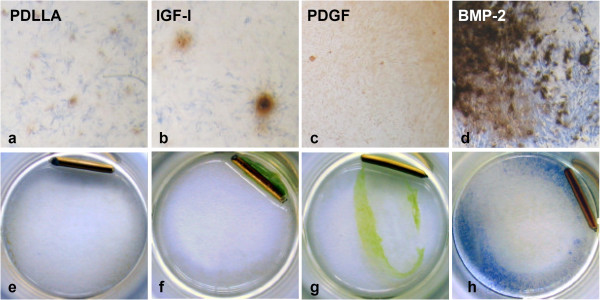
a-d) hOB 15 days after culturing with different growth factors stained with a combination of AP (blue) and v. Kossa. An intense mineralization is detectable in the osteoblast like cells treated with BMP-2 (d). e-h) C2C12 cells stained for alkaline phosphatase. The pluripotent mesenchymal cell line treated with BMP-2 (h) showed an intense blue alkaline phosphatase staining.

The results of the growth factor groups and the different assays were normalized to the PDLLA group which was set 100%. This method was chosen for accounting for differences between the serials. All cell cultures were started with a comparable cell number (day 0, Fig. 1a). Over the experimental period of 10 days a significant increase in cell number was detectable in the PDGFbb and IGF-I group compared to the PDLLA group (Fig. 1a). No effect of BMP-2 on cell proliferation was detectable. The enzymatic activity of alkaline phosphatase, however, was significantly higher in the BMP-2 treated osteoblast like cells at days 10 and 15 (Fig. 1b). The two other growth factors had no influence on the AP-activity. The von Kossa stain for mineralized extra cellular matrix after 15 days revealed a clear stimulating effect of BMP-2 on the mineralization (Fig. 2a–d). No effect on collagen-1 and osteocalcin synthesis was observed after treatment with growth factors (data not shown).

### C2C12 cell line

**Figure 3:**
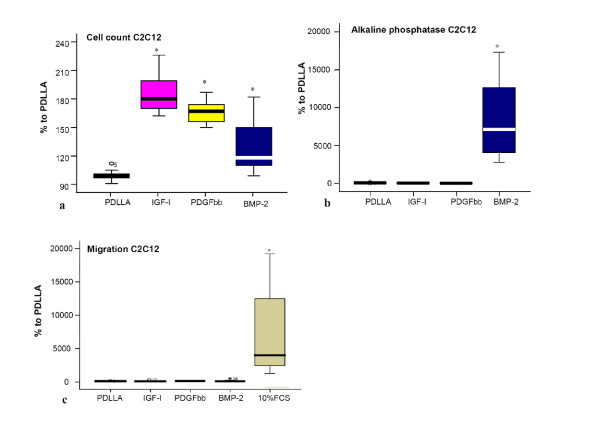
a) Cell count of the pluripotent mesenchymal cell line (C2C12) treated with different growth factors. The data presented are normalized to the control group (PDLLA) which is set 100%. A significant increase in the cell number was seen after treatment with PDGFbb, IGF-I or BMP-2 in comparison to the PDLLA treated cells (ANOVA, Dunnett). b) Alkaline phosphatase activity (AP) of pluripotent mesenchymal cell line (C2C12) treated with different growth factors. The data presented are normalized to the control group (PDLLA) which is set 100%. A significant increase in AP activity was seen after treatment with BMP-2 in comparison to the PDLLA treated cells (ANOVA, Dunnett). c) Migration assay of C2C12 cells. A significant migration was detectable in the control group (10% FCS) but not in the growth factor groups.

All three growth factors, PDGFbb, IGF-I and BMP-2, stimulated significantly the cell proliferation in the myoblast cell line (Fig. 3a). The effect of BMP-2, however, was less pronounced. The alkaline phosphatase activity was only significantly increased after stimulation with BMP-2 compared to the PDLLA group (Fig. 3b). This is also clearly visible in the alkaline phosphatase stain in Figure 2e–h.

In the standard cell culture wells, the pluripotent mesenchymal cell line showed an accumulation around IGF-I coated k-wire (Fig. 2g). This effect was not seen for PDGFbb or BMP-2.

The migration assays (Boyden Chamber) revealed a significant migratory effect of the positive control (FCS) on the cells. None of the growth factors, however, showed an effect on the migratory activity of the pluripotent mesenchymal cell line (Fig. 3c).

## Discussion

The biological stimulation of bone regeneration is a growing field. Several growth factors necessary for bone development, maintenance, and regeneration have been identified. This study aims to compare the effectiveness of three growth factors approved for clinical use released from a local drug delivery system: PDGFbb, IGF-I and BMP-2. Two different cell types were used to investigate the effect of the different growth factors. In both cell types, primary human osteoblast like cells and a murine pluripotent mesenchymal cell line, BMP-2 induced cell differentiation, whereas IGF-I and PDGFbb stimulated cell proliferation. None of the investigated growth factors induced migration in the Boydan chamber assay.

The pluripotent myoblast cell line is a well established system for testing osteoinductivity by using the reversible potential of the cells to differentiate into osteoblastic phenotype after stimulation with osteoinductive factors [18]. The observed effect of BMP-2 on pluripotent mesenchymal cell line is in accordance with previous studies showing the osteoinductivity of this growth factor [23,24]. The performed Boyden Chamber experiment showed no migratory effect of the used growth factors on the C2C12 cells and this is in accordance with a study by Allen at al. [22]. The proliferating effect was the strongest in the myoblast culture treated with IGF-I followed by PDGFbb and then BMP-2.

The mitogenic effect of the growth factors IGF-I and PDGFbb released from the implant coating on osteoblast like cells and pluripotent mesenchymal cells is also in accordance with previous studies [8,25,26]. The proliferating effect of the growth factors seem to be differentiation depending, because BMP-2 stimulated proliferation only in the pluripotent cell line, whereas no effect was shown on the osteoblast like cells.

The results concerning the effect of both factors (PDGFbb and IGF-I) on osteoblast differentiation are controversy. Some studies demonstrated an enhanced collagen and osteocalcin synthesis [8,27,28], other studies, however, found no effect [25,29]. The present study showed also no effect on the activity of alkaline phosphatase, the collagen-1 synthesis and the osteocalcin level in the medium. The stimulating effect of BMP-2 on alkaline phosphatase activity of osteoblast like cells has been reported earlier [30,31]. The stimulating effect of BMP-2 on osteocalcin expression as described by Spinella-Jaegle et al. was not seen in the present study [32]. This might be due to the different cells used in the experiments. Spinella-Jaegle performed the experiments with the murine preosteoblastic cell line MC3T3 and in the present study primary human osteoblast like cells were used.

*In vivo *studies on bone healing revealed an expression of the three analyzed growth factors at different healing phases. Cho and coworkers used a mouse fracture model and found BMP-2 expression only at the first day after fracture indicating the role in the very early healing phase [33]. The quantification of IGF-I during rat fracture healing on the protein level revealed no increase in the early phase in comparison to the unfractured tibia. In the phase corresponding to the chondrogenesis and intramembraneaus ossification (days 10, and 15) a significant increase of IGF-I was detectable [34]. The immunohistochemical detection of PDGF during mice fracture healing showed that PDGF is expressed by several cell types during almost the entire healing period [35].

The different phases of fracture healing are characterized by the presents of different cell types [36,37]. In addition, the receptors on the cells also vary depending on the differentiation stage of the cell [38,39]. These data point out that the three investigated factors are important during different healing phases. The controlled temporal regulation of growth factor action is necessary because of the interaction of the different factors. Less information on the interaction of factors is available, but the study by Cirri et al. demonstrated the inhibition of PDGF induced cell proliferation after application of insulin [26]. The simultaneous application of IGF-I, TGF-β1 and PDGF to osteoblast like cells enhanced the *in vitro *bone formation synergistically [40]. Therefore, for optimal stimulation of bone repair the controlled and local delivery of factors and factor combinations is mandatory [14,41,42]. In the present study the growth factors were delivered by using a local drug delivery system. Further studies are now necessary to identify the most potent stimulating factors and the timing of delivery. Based on the implant coating for local drug delivery we will develop a sequential drug release system for the temporally optimized delivery of stimulating factors.

## Conclusion

In conclusion, the growth factors IGF-I and PDGFbb delivered with a local drug delivery system stimulated cell proliferation, whereas BMP-2 showed a dramatic effect on differentiation in osteoblast precursor cells and osteoblast like cells.

## Competing interests

The author(s) declare that they have no competing interests.

## Authors' contributions

BW conceived, supervised, coordinated the study, performed the statistical analysis and wrote the manuscript. NB carried out the experiments with the C2C12 cells ML carried out the experiments with the osteoblast like cells. TV and GS participated in the study design and coordination and helped to draft the manuscript. All authors read and approved the final manuscript.
